# Postburn Auricular Mucormycosis in an Immunocompetent Patient: Early Diagnosis and Multidisciplinary Management

**DOI:** 10.7759/cureus.96835

**Published:** 2025-11-14

**Authors:** Shadi Shinnawi, Rafat Dawayme, Muhammad Zahlaka

**Affiliations:** 1 Otolaryngology - Head and Neck Surgery, UC Davis Health, Sacramento, USA; 2 Otolaryngology - Head and Neck Surgery, Rambam Health Care Campus, Haifa, ISR; 3 Internal Medicine, Hillel Yaffe Medical Center, Hadera, ISR; 4 Otolaryngology - Head and Neck Surgery, Wolfson Medical Center, Kafr Qara, ISR

**Keywords:** auricular trauma, burn injury, fungal infections, head & neck surgery, mucormycosis

## Abstract

Mucormycosis is a rare, rapidly progressive fungal infection that can cause devastating tissue destruction, particularly in vulnerable settings. We report a rare case of postburn auricular mucormycosis in a previously healthy, immunocompetent patient. After a motor vehicle accident that caused extensive burns, the patient developed dead tissue in both ears and pus in the ear discharge. A quick evaluation by an ear, nose, and throat (ENT) specialist, a bedside biopsy, and lab tests confirmed invasive mucormycosis. We promptly initiated systemic amphotericin B and performed extensive surgical debridement of all infected tissue. This approach resulted in sustained resolution of the infection, with no evidence of recurrence at subsequent follow-up. This case highlights the importance of being alert to this condition, diagnosing it early, and using a team approach to manage mucormycosis related to burns. Quick action is crucial to reduce the risk of death and improve functional and mental health outcomes in these difficult infections. Reports of auricular mucormycosis following burn injury in immunocompetent individuals are exceedingly uncommon, highlighting the clinical relevance of this case.

## Introduction

Mucormycosis is a rare and aggressive fungal infection that progresses quickly and has a high risk of death. The fungi responsible for this condition belong to the class Zygomycetes and the order Mucorales [1]. This life-threatening infection is known for invading blood vessels and lymphatics, causing blood clots, ischemia, and tissue necrosis [2]. Under the microscope, mucormycosis shows broad, non-septated hyphae that typically branch at right angles.

In its early stages, the disease can resemble other infections or necrotizing conditions, such as bacterial perichondritis, malignant otitis externa, or certain types of soft tissue cancers. This similarity can lead to delays in diagnosis. Mucormycosis usually affects people with weakened immune systems, but there is a growing understanding that Mucorales fungi can also cause severe wounds in healthy individuals, especially after burns, accidents, natural disasters, or combat injuries [3-5].

The infection can occur in various parts of the body, including the paranasal sinuses, rhino-orbital or rhino-orbito-cerebral areas, lungs, gastrointestinal tract, skin, and, more rarely, as part of a widespread infection [6]. Involvement of the outer ear is extremely rare, with only a few cases reported. Imaging studies often lack specificity, and fungal cultures may not be reliable; therefore, a definite diagnosis relies on histopathological confirmation [3].

The best management involves quickly addressing any underlying risk factors with systemic antifungal treatment, particularly amphotericin B, along with aggressive surgical debridement of the infected area [2]. Early detection and a coordinated, team-based approach are vital for improving outcomes. Even with prompt and appropriate treatment, the infection still has a high death rate. This report shares a very rare case of auricular mucormycosis following a burn, highlighting both the challenges in diagnosis and the positive results achieved through combined medical and surgical treatment.

## Case presentation

A 29-year-old previously healthy man was admitted to a secondary hospital after a serious motor vehicle collision with a subsequent vehicle explosion. Upon arrival, he had severe respiratory distress and required immediate intubation. Once stabilized, a CT scan of his entire body showed no injuries to his internal organs. However, because he had extensive burns covering 55% of his body, predominantly affecting the limbs, he was transferred to our specialized burn center that same day for intensive care.

In the burn ICU, he underwent several rounds of debridement and skin grafting procedures with the plastic surgery team. His condition steadily improved, and he remained stable, showing no signs of systemic infection. On hospital day 20, he developed discharge from his left ear, which suggested a localized infection and prompted a consultation with an ear, nose, and throat (ENT) specialist.

**Figure 1:**
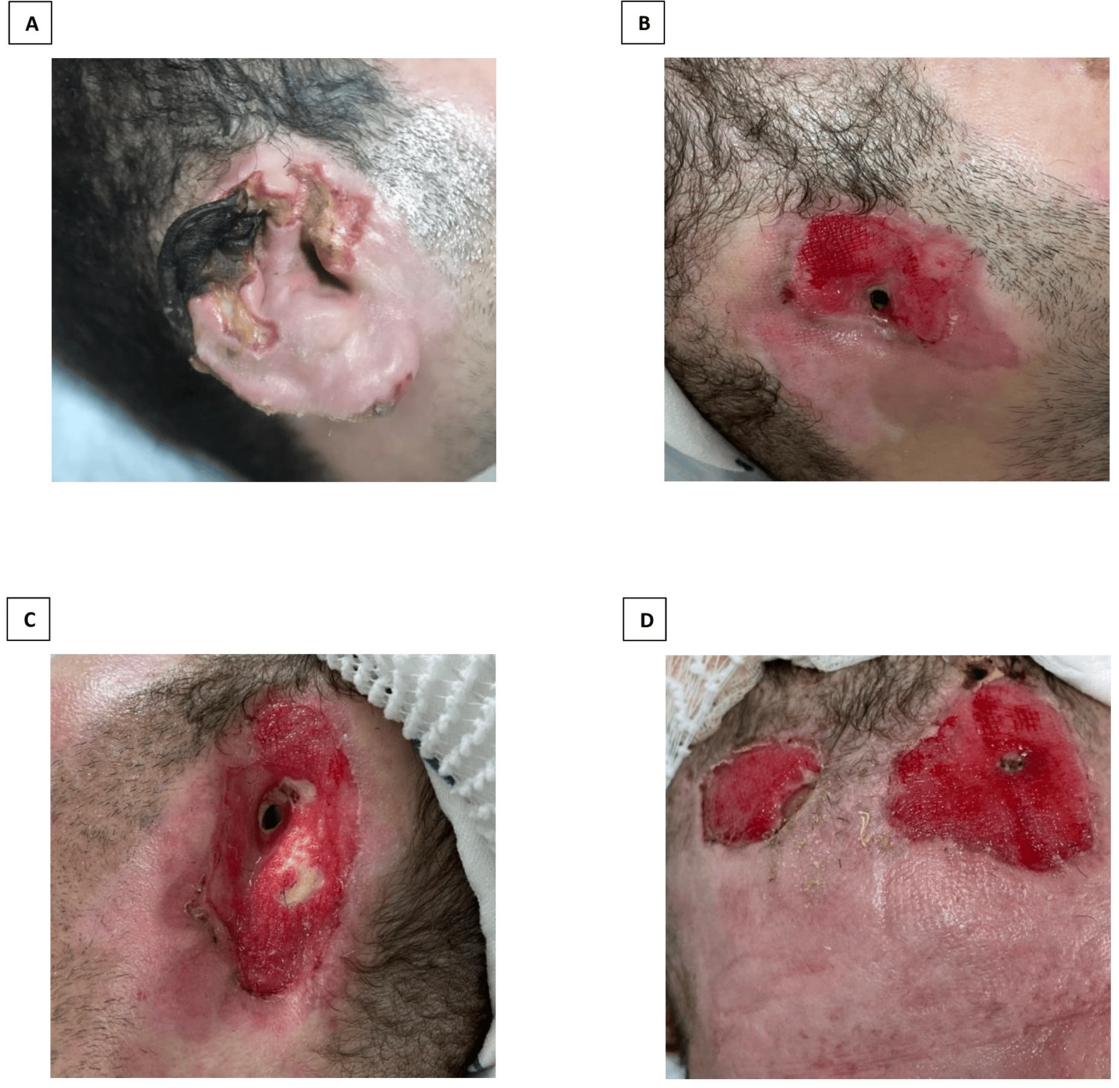
(A) Patient’s right ear upon presentation. (B) Patient’s right ear at day 60 postoperatively. (C) Patient’s left ear at day 60 postoperatively. (D) Patient’s forehead at day 60 postoperatively.

**Figure 2:**
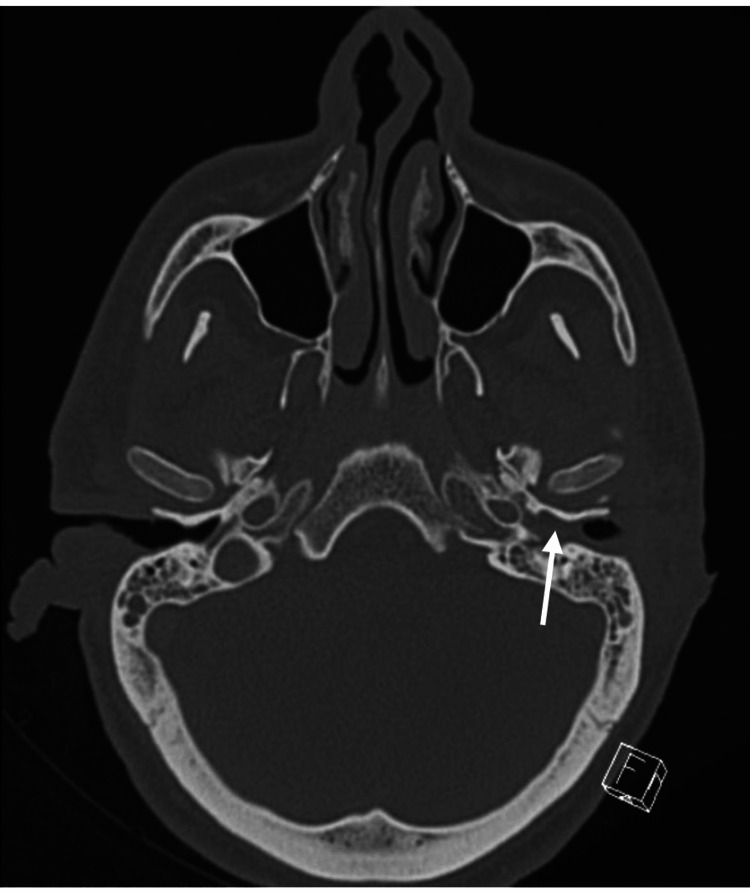
Preoperative CT scan with contrast showing opacity in left canal (white arrow), middle ear cavity, and mastoid cavity without any bone destruction.

The ENT examination showed that the patient did not have a fever and was stable. Both ears displayed moist gangrenous changes and dark discoloration (Figure 1). When the left ear was examined with an otoscope, there was notable swelling and almost complete blockage of the external auditory canal. The nasal and oral cavities were unaffected; however, two necrotic lesions were noted on the forehead. A CT scan showed opacification in the left external auditory canal, middle ear, and mastoid air cells, with no signs of bone erosion (Figure 2). A CT scan was chosen because it assesses bone erosion and involvement. This helps distinguish invasive fungal infection from other conditions like necrotizing otitis externa. MRI is more sensitive for soft tissue, but it is not always easily available in acute settings.

Given the high mortality associated with delayed diagnosis of mucormycosis and its key features, including tissue death, rapid progression, and necrotic eschar, plus the patient’s immunocompetent state after the trauma, urgent diagnostic and treatment steps were initiated. Major burns can cause temporary immune dysfunction; immunocompetent here refers only to the absence of chronic immunosuppression. The team focused on identifying the fungus early. A bedside biopsy of the damaged ear cartilage was done, and samples were sent for histopathology and ear discharge cultures.

**Figure 3:**
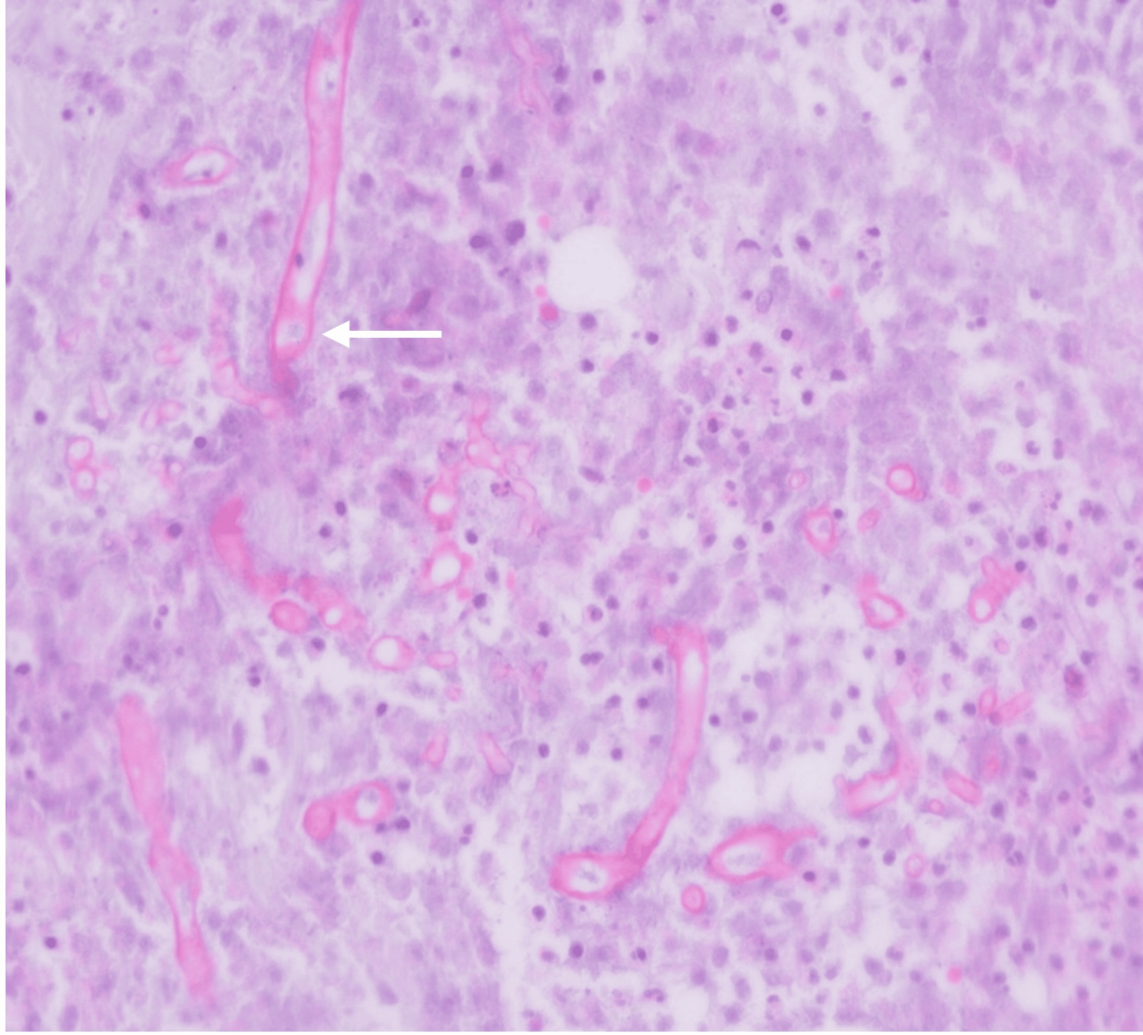
Broad aseptate hyphae (white arrow), typical for those species belonging to the Mucorales, hematoxylin and eosin (H&E) stain, original magnification ×40.

Microscopy of the frozen section showed broad, non-septated fungal hyphae with right-angle branching invading the cartilage (Figure 3), consistent with mucormycosis. Pus culture from the left ear discharge showed growth of a fungus from the Mucorales group, specifically Lichtheimia, further corroborating the diagnosis. Systemic antifungal treatment with intravenous liposomal amphotericin B (5 mg/kg/day) was initiated immediately and continued for three weeks. Aggressive surgical debridement was planned on the same day as the frozen section biopsy, allowing for immediate intervention.

Under general anesthesia, both auricles and the cartilaginous portions of the external auditory canals were removed, and the forehead lesions were debrided to bleeding viable margins. Exploring the middle ear revealed no complications beyond the external canal. Intraoperative final histology confirmed the invasive mucormycosis diagnosis.

After surgery, the wounds were managed with open dressings. Daily hydrogen peroxide irrigation and topical amphotericin B were used to reduce the fungal load. Repeat biopsies during dressing changes showed no remaining fungus. The systemic antifungal therapy continued for weeks while closely monitoring kidney function and electrolytes.

The patient stayed stable and showed no systemic spread; laboratory results remained normal. By postoperative day 60, he had recovered from the acute fungal infection (Figure 1). No recurrence of the fungus occurred as the wounds healed by secondary intention, and new tissue filled the cleaned areas. Using Ivalon® sponge tampons helped prevent narrowing of the external auditory canal.

The patient faced significant psychosocial challenges due to the removal of both ears. Multidisciplinary support included plastic surgery and mental health counseling, with plans for future ear reconstruction.

This case emphasizes the critical need for early suspicion, teamwork in care, and collaborative specialist interventions in successfully treating burn-related mucormycosis. It highlights the importance of rapid diagnosis, aggressive surgical procedures, prompt antifungal treatment, and ongoing support for rehabilitation and psychosocial recovery.

## Discussion

Mucormycosis is a life-threatening fungal infection, with reported mortality rates ranging between 23% and 100% [7]. It is the third most common invasive fungal infection, following candidiasis and aspergillosis [6,8,9]. The fungi that cause this disease are found everywhere in the environment, including in soil, air, decaying organic matter, and spoiled food. Infections are rare in people with healthy immune systems but mainly affect those who are immunocompromised. The highest-risk groups include individuals with uncontrolled diabetes and those receiving prolonged systemic corticosteroid therapy.

Patients with severe burn injuries are especially at risk for cutaneous mucormycosis. The first cases were described in 1961 by Rabin et al. [10]. Later reviews have noted burn-related cutaneous disease, usually showing up as localized or deep infections, while widespread disease is seen less often [4]. These infections progress quickly and lead to tissue death, but they tend to have a better outlook than other types of mucormycosis, with about 31% mortality compared to 50-80% [11]. Involvement of the ear is very rare, and this case is the first reported instance in English literature of invasive mucormycosis affecting auricular cartilage after a thermal injury.

The exact way the fungi enter the auricular cartilage is unclear. In this instance, we hypothesize that direct inoculation happened when the patient rolled in soil to put out flames, which has been noted in other cases of burn-related mucormycosis [12].

Diagnosis relies on a combination of high clinical suspicion, imaging, culture, and histopathologic confirmation [10]. A firm diagnosis is typically made by directly showing the fungus in tissue or biopsy samples [9]. Standard treatment rests on three main steps: (1) extensive surgical debridement to lower the fungal load and improve antifungal drug penetration, (2) systemic antifungal therapy, and (3) addressing any underlying issues in immunocompromised patients [13] Amphotericin B is the preferred treatment, especially the liposomal version (AmBisome®, Gilead Science, Foster City, CA), because it has less impact on the kidneys and allows for higher doses.

Research by Chamilos et al. indicates that starting amphotericin B quickly is linked to better survival; starting within five days of diagnosis showed survival rates of 83%, compared to 49% for those who started treatment later [14]. Therefore, rapid surgical and medical treatment is crucial.

A review by Devauchelle et al. outlined the trends of burn-related mucormycosis, noting that burns usually cover 42.5-65% of total body surface area (TBSA) and mortality rates range from 29% to 100% [5]. Kyriopoulos et al. also examined six trauma-related cases (TBSA 45-71%) and emphasized the need for early detection, wide debridement, and amphotericin therapy for suspected mold infections in burn wounds [12].

In our patient, intravenous liposomal amphotericin B was started as soon as there was clinical suspicion, and culture results later confirmed the diagnosis. Surgical debridement occurred the following day. Additionally, topical amphotericin and hydrogen peroxide dressings were applied twice daily until healthy granulation tissue appeared, aligning with previous studies that recommended hydrogen peroxide for open mucormycosis lesions [15].

While rhinocerebral mucormycosis in immunocompromised patients is well known in otolaryngology, invasive fungal infections after burn injuries are much less common. This case highlights the need for a high index of suspicion, as prompt diagnosis and treatment are directly linked to improved survival.

A major limitation of this report is the relatively short follow-up period (60 days). Longer-term data on recurrence are not available and should be addressed in future studies.

## Conclusions

Mucormycosis is a rare but serious infection in burn patients. It usually appears as a skin infection at the burn site. Managing it is difficult and requires high clinical suspicion, quick diagnosis, removal of dead tissue, and immediate antifungal treatment. Amphotericin B is the preferred treatment. Soil molds can enter burn wounds at the time of injury, making it important to consider the cause of the burn when assessing the risk of mucormycosis. Burns to the ears need special care because their exposed shape makes them more likely to come into contact with contaminated ground, increasing their risk for fungal infection and complications.
